# Virome analyses of *Hevea brasiliensis* using small RNA deep sequencing and PCR techniques reveal the presence of a potential new virus

**DOI:** 10.1186/s12985-018-1095-3

**Published:** 2018-11-26

**Authors:** Paula L. C. Fonseca, Fernanda Badotti, Tatiana F. P. de Oliveira, Antônio Fonseca, Aline B. M. Vaz, Luiz M. R. Tomé, Jônatas S. Abrahão, João T. Marques, Giliane S. Trindade, Priscila Chaverri, Eric R. G. R. Aguiar, Aristóteles Góes-Neto

**Affiliations:** 1 Department of Microbiology, Instituto de Ciências Biológicas, Universidade Federal de Minas Gerais (UFMG), Belo Horizonte, MG 31270-901 Brazil; 2 Department of Chemistry, Centro Federal de Educação Tecnológica de Minas Gerais (CEFET-MG), Belo Horizonte, MG 30421-169 Brazil; 3 LANAGRO/MG –Laboratório Nacional da Agricultura, Ministério da Agricultura (MAPA), Pedro Leopoldo, MG 33600-000 Brazil; 4 Faculdade de Minas (FAMINAS), Belo Horizonte, MG 31744-007 Brazil; 5 Department of Biochemistry and Immunology, Instituto de Ciências Biológicas, Universidade Federal de Minas Gerais (UFMG), Belo Horizonte, MG 31270-901 Brazil; 6 Department of Plant Science and Landscape Architecture, University of Maryland, College Park, MD 20742 USA; 7 Escuela de Biología, Universidad de Costa Rica, San Pedro, San José, 11501-2060 Costa Rica; 8 Instituto de Ciências da Saúde, Universidade Federal da Bahia (UFBA), Salvador, BA ,40110-100 Brazil

**Keywords:** *Hevea brasiliensis*, Virome, RNA deep sequencing, ddPCR, HBrV

## Abstract

**Background:**

*Hevea brasiliensis* is an important commercial crop due to the high quality of the latex it produces; however, little is known about viral infections in this plant. The only virus described to infect *H. brasiliensis* until now is a *Carlavirus*, which was described more than 30 years ago. Virus-derived small interfering RNA (vsiRNAs) are the product of the plant’s antiviral defense triggered by dsRNA viral intermediates generated, during the replication cycle. These vsiRNAs are complementar to viral genomes and have been widely used to identify and characterize viruses in plants.

**Methods:**

In the present study, we investigated the virome of leaf and sapwood samples from native *H. brasiliensis* trees collected in two geographic areas in the Brazilian Amazon. Small RNA (sRNA) deep sequencing and bioinformatic tools were used to assembly, identify and characterize viral contigs. Subsequently, PCR amplification techniques were performed to experimentally verify the presence of the viral sequences. Finally, the phylogenetic relationship of the putative new virus with related viral genomes was analyzed.

**Results:**

Our strategy allowed the identification of 32 contigs with high similarity to viral reference genomes, from which 23 exhibited homology to viruses of the *Tymoviridae* family. The reads showed a predominant size distribution at 21 nt derived from both strands, which was consistent with the vsiRNAs profile. The presence and genome position of the viral contigs were experimentally confirmed using droplet digital PCR amplifications. A 1913 aa long fragment was obtained and used to infer the phylogenetic relationship of the putative new virus, which indicated that it is taxonomically related to the Grapevine fleck virus, genus *Maculavirus*. The putative new virus was named *Hevea brasiliensis virus* (HBrV) in reference to its host.

**Conclusion:**

The methodological strategy applied here proved to be efficient in detecting and confirming the presence of new viral sequences on a ‘very difficult to manage’ sample. This is the second time that viral sequences, that could be ascribed as a putative novel virus, associated to the rubber tree has been identified.

**Electronic supplementary material:**

The online version of this article (10.1186/s12985-018-1095-3) contains supplementary material, which is available to authorized users.

## Background

*Hevea brasiliensis* (the rubber tree) is described as source of higher quality latex and rubber among all known plant species. The latex produced by this plant has a set of features such as elasticity, abrasion and impact resistance, heat dispersion and malleability, which makes it the ideal feedstock for building products in many different areas, such as engineering, medical and pharmaceutical industries [1, 2]. To date, no synthetic rubber obtained from petroleum has shown similar properties, thus making *H. brasiliensis* latex irreplaceable [3]. Natural rubber is an indispensable commodity used to manufacture more than 50,000 products, accounting for over US$16.5 billion annually in global exportation [4].

Currently, more than 90% of natural rubber production occurs in Asia, mainly in Malaysia, Thailand and Indonesia, even though the rubber tree originated in the Brazilian Amazon [5]. Commercial rubber plantations in Brazil and other countries in Latin America have failed due to the South American Leaf Blight (SALB) disease [6], caused by the fungus *Pseudocercospora ulei* [7]. Apart from SALB, *H. brasiliensis* is also known to be susceptible to many other diseases caused by fungi [8], such as anthracnose caused by *Colletotrichum gloeosporioides* [9] or the powdery mildew caused by *Oidium hevea* [10]. Virus have also been described to infect rubber trees. However, the knowledge about viral pathogens is restricted to one species belonging to the genus *Carlavirus*, which is associated with the Leaf Disease of Viral Origin [11].

In response to viral infections, eukaryotic organisms have developed different strategies to protect their genomes. RNA interference (RNAi) is an important regulatory mechanism to induce the silencing of self and non-self RNA through sequence-specific homologous interactions [12]. In this process, small RNAs (sRNAs) are generated through the processing of long double-stranded RNA (dsRNA) precursors by an RNAse III-like enzyme (DICER). The produced short sequences are loaded onto the argonaute enzyme (AGO) to generate the RNA-induced silencing complex (RISC), which will target complementary regions of mRNA, leading to translation inhibition and mRNA destabilization [12–14]. In plants, this RNA interference pathway is named Post-Transcriptional Gene Silencing (PTGS) [15], and small interfering RNAs (siRNAs) are the most important sRNAs in the plant’s response against viral infections [16].

Therefore, the sequencing of sRNAs is a useful tool for detecting phytoviruses, also in asymptomatic and infected plants [17, 18]. The deep sequencing of virus small interfering RNAs (vsiRNAs) can be used to reconstitute the sequences from which they originated and can also be indicative of an infection without requiring direct detection of the virus. This strategy has been successfully applied to identify and characterize viruses in plants, fungi and animals [19–21].

Here, we investigated the virome of asymptomatic native *H. brasiliensis* trees using sRNA deep sequencing, bioinformatics tools and PCR techniques. This strategy allowed to identify viral sequences, which after their assembly, were found to be phylogenetically related to Grapevine fleck virus (GFkV), genus *Maculavirus*. Our findings expand the knowledge about the *H. brasiliensis* virome and suggest that other studies are necessary to explore the viral biodiversity in this plant.

## Methods

### Study areas and sample collection

**Fig. 1:**
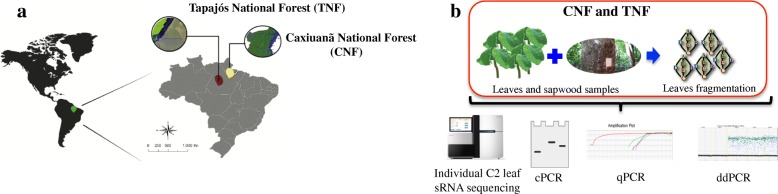
**a** Geographic location in the Brazilian Amazon where the *Hevea brasiliensis* samples were collected. The Tapajós National Forest (TNF) is indicated in red and the Caxiuanã National Forest (CNF) in yellow. **b** Simplified scheme showing leaf collection, fragmentation, sRNA sequencing and PCR amplifications by cPCR, qPCR and ddPCR

This study was carried out in two different areas in the state of Pará, Brazil: Caxiuanã National Forest - CNF (01°37’S – 02°15’S; 51°19’W – 51°58’W) and Tapajós National Forest - TNF (02°45’S–04°15’S; 54°45’W–55°30’W) (Fig. 1a). Native *H. brasiliensis* species prevail in these areas, which are characterized by a short dry season and excessive precipitation, with a mean annual temperature of 25.9 °C and 82% humidity. The permissions for the study were obtained from the *Sistema de Autorização e Informação em Biodiversidade* (SISBIO), authentication code 31162617.

Leaf and sapwood samples were randomly collected from five adult individuals in CNF (C1-C5) and from 10 individuals in TNF (T1 – T10), from which five were adults and five were plantlets. All collected samples were originated from asymptomatic plants with the leaves showing homogenous green coloration without any kind of discoloration, wilting, or necrotic lesions. Five fragments from each leaf (Fig. 1b) and five pieces (5 mm each, 100 mg in total) of sapwood fragments (3–6 cm periderm) from each individual were placed in sterile cryotubes containing 1.0 mL of RNAlater® Stabilization Solution (Thermo Fisher Scientific, Carlsbad, CA, USA), transported to the laboratory in an ice box, and stored at − 80 °C [22].

### RNA extraction and small RNA deep sequencing

Leaf fragments from CNF were ground with liquid nitrogen and 500 mg aliquots were used for RNA extraction using TRIzol Reagent® (Thermo Fisher Scientific, Carlsbad, CA, USA) following the manufacturer’s recommendations. The quality and quantity of RNA were evaluated using spectrophotometry (NanoDrop ND-1000, NanoDrop Technologies, Wilmington, DE, USA) and automated electrophoresis systems (2100 Bioanalyzer, Agilent RNA 6000 Nano Kit, Agilent Technologies, Waldbronn, DE).

RNA samples were stored with 30 μL of RNA protection reagent (OMEGA bio-tek, Norcross, GA, USA). The samples were prepared for sequencing with the NEXTflex Small RNA – Seq Kit V3 and sRNAs were selected by size (15–35 nt) in denaturing SDS-PAGE electrophoresis (Bio Scientific Corp, Austin, TX, USA) and sequenced using Illumina HiSeq (Illumina, San Diego, CA, USA).

### Bioinformatic analyses

Pre-processing of sRNA libraries and virome analyses were performed as described by [23]. Briefly, raw sequences were submitted to quality filters and adaptor removal. Sequences with low Phred quality (< 20), ambiguous nucleotides and/or a length shorter than 15 nt were eliminated. The remaining sequences were mapped and compared to all bacterial, fungal and *H. brasiliensis* reference sequences available on the National Center for Biotechnology Information - NCBI (https://www.ncbi.nlm.nih.gov/) using Bowtie, allowing one mismatch [24]. The *H. brasiliensis* genome was downloaded from the Genome Online Database – GOLD (https://gold.jgi.doe.gov).

Sequences that did not present similarities with bacteria, fungi or the host were used for contig assembly and subsequent analyses. Assembled contigs greater than 50 nt were characterized based on sequence similarity and pattern-based strategies. The identification of conserved domains was performed using HMMER [25]. The sRNA size profile was calculated as the frequency of each sRNA read size on the reference genome, considering each polarity separately. The density of sRNAs was calculated as the number of times sRNA reads covered each nucleotide on the reference sequence genome. The Z-score was used to normalize the sRNA size profile and to plot heat maps for each contig or reference sequence using the R with ggplots package [26]. Pearson correlation (confidence interval > 95%) of the Z score values was used to estimate the relationship between sRNA profiles from different contigs and reference sequences. Similarities between sRNA profiles were computed using hierarchical clustering with UPGMA as the linkage criterion. Groups of sequences with more than one element with at least 0.8 of Pearson correlation between each other were assigned to clusters. The sRNA size profile coverage density was calculated using *in-house* Perl scripts and plotted using the R program with the package ggplot2 [23].

To predict the genome organization of the viral assembled contigs, we developed a strategy based on sequence similarity searches. First, we compared the contigs against the non-redundant GenBank database (NR) using Blast (minimum *e*-value of 1e^− 5^) to select the closest references. Once the genome viral references were selected (Additional file 1: Figure S1), the contigs positioning in each genome were calculated as the total number of contigs mapping into the reference. A ‘partial score’ was generated for each contig against each reference and stored (Additional file 1: Figure S1), subsequently a ‘cumulative score’ was generated (Additional file 1: Figure S1) considering the sum of all the ‘partial scores’.

### Detection of viral RNA by qPCR and ddPCR

To confirm the pipeline used for contig ordering, conventional PCR (cPCR), quantitative PCR (qPCR) and digital droplet PCR (ddPCR) amplification techniques were performed using oligonucleotides designed to amplify four larger fragments (Additional file 2: Table S1).

A total of 200 ng of total RNA were used in Reverse Transcriptase Reaction (RT-PCR) with random primers (500 ng/uL) (Thermo Fisher Scientific, Carlsbad, CA, USA) and M-MLV Reverse Transcriptase (Promega, Madison, WI, USA), following the protocols described by [27]. Conventional PCR was performed using 1.5 μL of each designed primer (10 pmol/μL), 4.0 μL (200 ng) of cDNA using KAPA Taq PCR Kit (KAPA BioSystems, Wilmington, MA, USA). The amplification reaction was performed using the annealing temperature at 56 °C. The cDNA samples were also used to amplify the *H. brasiliensis* actin constitutive gene by qPCR to evaluate the extraction efficiency [28].

The fragment amplification by qPCR was performed using TaqMan Universal Master Mix II (Thermo Fisher Scientific, Carlsbad, CA, USA) in a solution containing 2.0 μL (100 ng) of cDNA in a reaction volume of 8.5 μL containing 5.0 μL of TaqMan Universal Master Mix II (Thermo Fisher Scientific, Carlsbad, CA, USA) and 1.5 μL of primers (10 pmol/μL) with probes (5 pmol/μL), and 2.0 μL of DNase/RNase-free water. Amplification reactions were performed according to the TaqMan recommendations (Thermo Fisher Scientific, Carlsbad, CA, USA) with an annealing temperature of 60 °C. After amplification, the qPCR products were cleaned up with a GenElute PCR Clean-up Kit (Sigma-Aldrich, St. Louis, MO, USA) and sequenced using traditional Sanger technology. The ddPCR was carried out using ddPCR Supermix for Probes (BIO-RAD, Hercules, CA, USA), 2.0 μL (100 ng) of cDNA in a reaction volume of 18.0 μL containing 12.5 μL of 2Xand ddPCR Supermix for Probes (BIO-RAD, Hercules, CA, USA), 1.5 μL of primers (10 pmol/ μL) with probes (5 pmol/ μL), and 4 μL of DNase/RNase-free water. The droplet was transferred to a semi-skirted 96-well PCR plate (Eppendorf, Hamburg, DE), which was sealed and subjected to amplification in a Px2 Thermal Cycler (Thermo Electron Corporation, Foster City, CA, USA). Following PCR amplification, the plate was placed in a QX200 droplet reader (BIO-RAD, Hercules, CA, USA). The amplicon quantity was evaluated using QuantaSoft version 1.7 (BIO-RAD, Hercules, CA, USA) by determining the threshold value and the number of positive copies of the target in a 20 μL reaction.

### Phylogenetic analysis

The viral contigs were translated into amino acids, concatenated and aligned with reference sequences of the *Tymoviridae*, *Alphaflexiviridae, Gammaflexiviridae* and *Betaflexiviridae* families using MAFFT version 7 using the G-INS–i criteria. Viral sequences of the referred families were obtained from NCBI protein databases under the following accessions: *Tymoviridae* family: Grapevine fleck virus (NP_542612); Citrus sudden death-associated virus (YP_224218); Mayze rayado fino virus (AAK52838); Oat blue dwarf virus (NP_044447); Anagyris vein yellowing virus (YP_002308578); Diascia yellow mottle virus (YP_002048673), Eggplant mosaic virus (NP_040968); Nemesia ring necrosis virus (YP_002308442); Ononis yellow mosaic tymovirus (NP_041257); Plantago mottle virus (YP_002308445); Turnip yellow mosaic virus (AAB2649); *Alphaflexiviridae* family: Shallot virus X (NP_620648); Botrytis virus X (AAL17722); Lolium latent virus (YP_001718499); Indian citrus ringspot virus (NP_203553); Potato virus X (YP_002332929); Sclerotinia sclerotiorum debilitation-associated RNA virus (YP_325662); *Betaflexiviridae* family: Apple stem grooving virus (NP_044335); Cherry virus A (NP_620106); Aconitum latent virus (NP_116487); Citrus leaf blotch virus (NP_624333); Apple stem pitting virus (NP_604464); Grapevine rupestris stem pitting-associated virus (NP_047281); Peach chlorotic mottle virus (YP_001497153); Apple chlorotic leaf spot virus (NP_040551); Peach mosaic virus (YP_002308565); Grapevine virus A (NP_619662) and *Gammaflexiviridae* family: Botrytis virus F (NP_068549).

Phylogenetic analyses were carried out in the Geneious 9 and MEGA 7 software using distance-based (distance matrix) and character-based (maximum likelihood) methods, respectively. Mean distances and the neighbor-joining algorithm were used for distance analyses, and the best-fit model of protein evolution, previously selected in ProtTest 3.2 with Akaike Information Criterion (AIC), was used for maximum likelihood analyses [29, 30]. Clade robustness was assessed using bootstrap proportions (1000 replicates). The trees were edited using FigTree 4.0 (http://tree.bio.ed.ac.uk/software/figtree/) and midterm rooted.

## Results

### Virome analyses

To investigate the virome of *H. brasiliensis*, one leaf sample obtained from one tree was selected to construct two sRNA libraries based on RNA quality. The two libraries were pooled, totalizing 21,446,061 reads. Reads with low quality or containing ambiguous bases were excluded, which resulted in 18,591,249 reads left. The pre-processing of the data resulted in a decrease of only ~ 13% of the reads, highlighting the representativeness and quality of our libraries. We enriched reminiscent reads for viral sequences by filtering out reads matching bacterial, fungal and host genomes. The filter step reduced the number of reads to 1,737,014, which were then used for contigs assemblage and characterization of the putative viral genome (Additional file 3: Table S2).

**Fig. 2:**
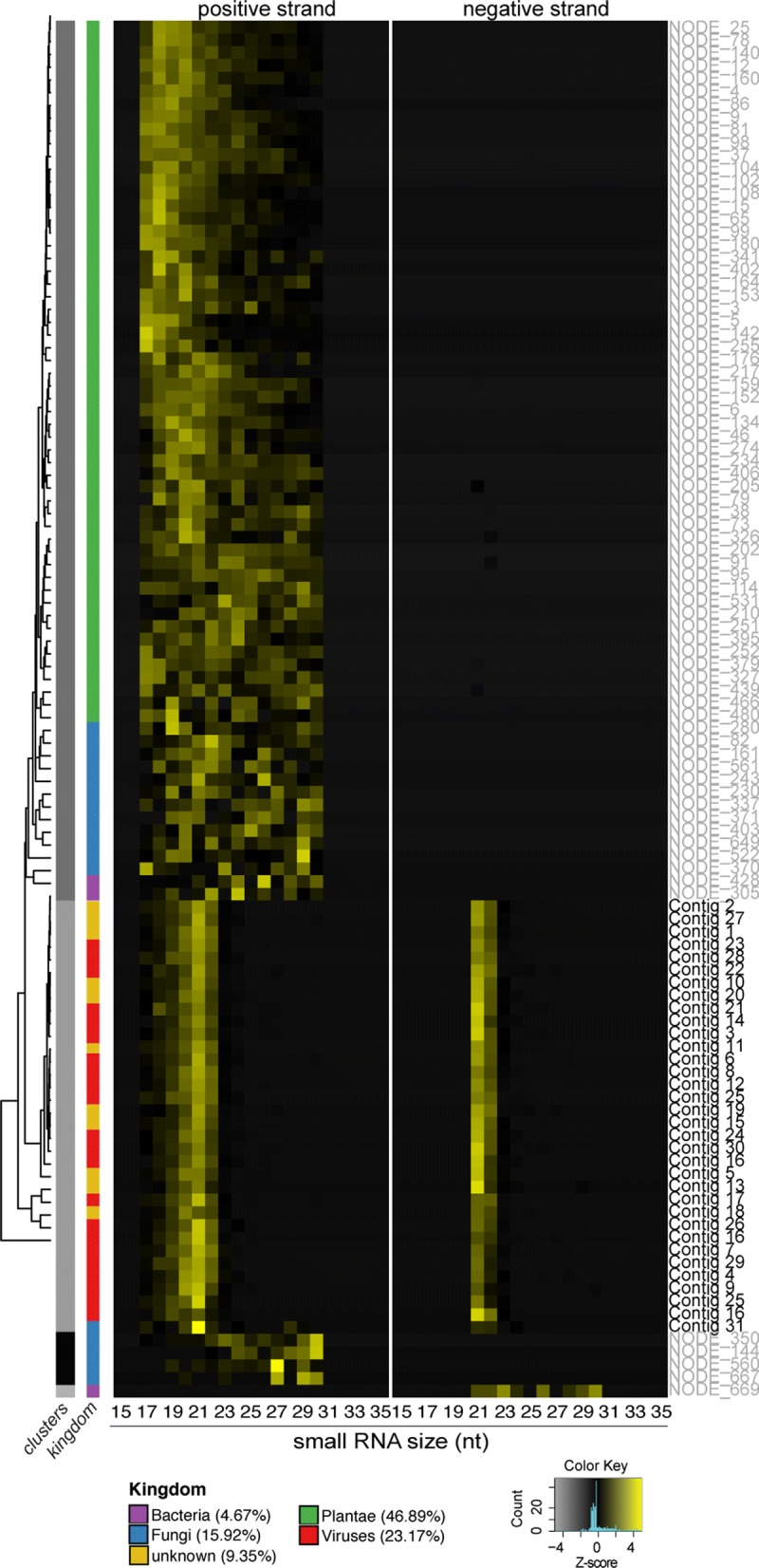
Heat map showing hierarchical clustering of small RNA size profiles of viruses, bacteria, plant and fungi contig sequences assembled for the *Hevea brasiliensis* leaf sample. Viral contigs with the same size profile in the positive and negative RNA strands are indicated on the right

A total of 110 non-redundant contigs were obtained and characterized through sequence similarity searches against NCBI databases. From these, 32 contigs showed significant similarity to the viral reference genomes, from which 23 exhibited homology at the protein level to positive sense single stranded RNA (ssRNA) viruses of *Tymoviridae* family. The remaining contigs that did not show similarity to the viral reference databases presented small RNA size distribution with predominance of 20–22 nt in length derived from both strands (Fig. 2, Additional file 4: Figure S2). We still detected contigs showing similarity with plant, bacteria and fungi, which likely represent sequences that are not present in the reference genomes deposited in NCBI database.

### Characterization of the viral genome

The investigation of the molecular characteristics of the sRNAs mapped into assembled contigs showed that most of the sequences with similarity to Plantae, Bacteria and Fungi have a broad size distribution and originated mostly from positive sense strand RNAs. On the other hand, small RNAs mapped into contigs exhibiting similarity to viral sequences showed a size distribution between 20 and 22 nt derived from both strands, which was consistent with the signature of sRNAs derived from vsiRNA pathway (Fig. 2).

**Fig. 3:**
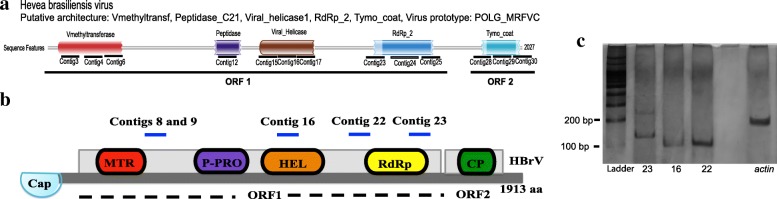
Genomic characterization of the putative *Hevea brasiliensis* virus (HBrV) as well as contigs amplification. **a** Contigs assigned into the domains of the replicase polyprotein: MTR, PRO, HEL, RdRp (ORF1) and the coat protein (ORF3). **b** Probable genomic organization of HBrV. Two ORFs were shown based on the viral contigs assembled by the sRNA sequencing generating a 1913 aminoacid sequence. Contigs 8 and 9, 16, 22 and 23 were amplified by qPCR and ddPCR. **c** Electrophoresis showing three fragments amplified by qPCR

The assembled contigs showed similarities to eleven different viral genomes of the *Tymoviridae* family. In addition, 13 out of 32 contigs presented conserved domains found in viruses of the *Tymoviridae* family (Fig. 3a). Contig sequences and details about the sequence similarity searches are shown in Additional file 5: Table S3 and Additional file 6: Table S4, respectively.

### Detection of viral RNA by qPCR and ddPCR

To confirm the presence and validate the predicted position of viral contigs, we designed oligonucleotides to amplify four fragments (Fig. 3b and red arrows in Additional file 4: Figure S2). Quantitative PCR (qPCR) was performed and successfully amplified three out of the four fragments (Fig. 3c), while ddPCR allowed the detection and quantification of the four viral fragments with RNA copies ranging from 25,125 to 104,250.

The presence of viral sequences was evaluated in all the *H. brasiliensis* leaves and sapwood samples collected in TNF and CNF areas using conventional PCR (cPCR), qPCR and ddPCR techniques. However, apart from the leaf sample from CNF, no signal of viral contigs was detected in the samples.

### Phylogenetic analyses

**Fig. 4:**
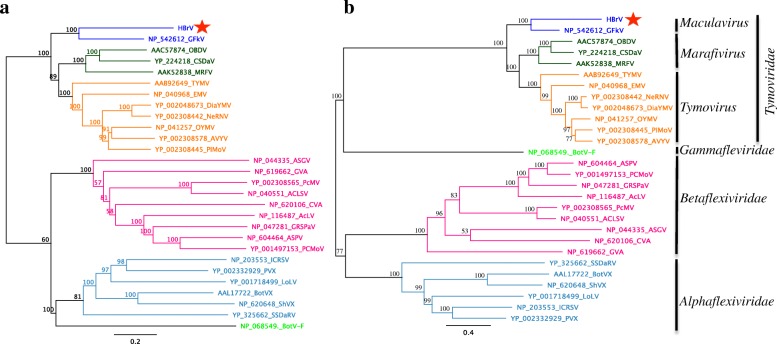
Phylogenetic analyses of the putative *Hevea brasiliensis* *virus* (HBrV) using **a** distance (Neighbor Joining) and **b** character-based methods (Maximum Likelihood). Stars indicate the HBrV position in the tree. Distinct taxa (genera or families) are shown in different colors

Based on the contig ordering strategy, we generated a 1913-aa long concatenated sequence that was used to infer the phylogenetic relationships of the putative virus. Using the distance-based method with the Neighbor-joining algorithm, it consistently clustered with the Grapevine fleck virus (GFkV) *Maculavirus* with strong statistical support (bootstrap = 100%). In addition, the putative virus was also phylogenetically close to *Marafivirus* and *Tymovirus* (bootstrap = 77.9%) (Fig. 4a). Maximum likelihood character-based phylogeny exhibited a similar topology, with the contig and GFkV *Maculavirus* clustering together (bootstrap = 100%). This analysis also revealed that the contig was related to *Marafivirus* and *Tymovirus* (bootstrap = 82%) (Fig. 4b). Altogether, distance- and character-based phylogenies indicate that the putative new virus belongs to a species closely related to *Maculavirus* and was named *Hevea brasiliensis* virus (HBrV) in reference to its host.

## Discussion

Viral dsRNA is used by the plant’s immune system to produce virus small interfering RNAs (vsiRNAs) 20–22 nucleotides in length [12, 13]. The vsiRNA class is usually the most abundant in plants infected with RNA virus and have been widely used as indirect evidence of the presence of viral infections since they are produced through recognition of viral dsRNA generated during the virus replication cycle [17, 23]. Therefore, our results indicate that the putative new virus HBrV is replicating in *H. brasiliensis* leaf, since the predominant size of reads entering in the viral contigs assembly was  20-22 nt in both strands.

Metagenomic studies usually generate fragmented sequences, which hamper the characterization of genome structure and phylogenetic analyses [31, 32]. To overcome this limitation, we developed an in silico strategy based on sequence similarity searches to define the order of viral contigs and their genome structure. Our analyses revealed that 32 contigs have high similarity to viral reference sequences, and 13 of them were assigned to five conserved protein domains found in viruses from the *Tymoviridae* family: methyltransferase (Mtr), RNA helicase (Hel), papain-like cysteine protease (P-Pro), RNA-dependent RNA polymerase (RdRp), and the coat gene.

The ordering and characterization of the viral contigs allowed the design of specific oligonucleotides to experimentally confirm the presence of the viral RNA sequences in our sample via PCR amplifications. Conventional PCR was not suitable to detect their presence, while using qPCR three out of four contigs were amplified. Small amounts of target molecules in a qPCR reaction could decrease amplification efficiency due to the reduced probability of binding oligonucleotides in the template [33]. Digital droplet PCR (ddPCR) allowed the amplification and absolute quantification of the four viral fragments covering five contigs. The improved performance of ddPCR is probably explained by the massive sample partitioning, high resistance to inhibitory effects of different matrices and the direct quantification that dispenses calibration curves [34, 35].

The use of in silico similarity searches associated with experimental confirmation of the RNA viral sequences allowed us to generate a 1913 aa long fragment, which was used to estimate the phylogenetic relationships of the putative virus. Distance and character-based phylogeny methods indicate that HBrV clusters with the Grapevine fleck virus, genus *Maculavirus* (*Tymoviridae* family).

Genomes of viruses belonging to the *Tymoviridae* family differ in the number of ORFs (from 1 to 4) and in the distribution of genes, but they all encode a large polyprotein essential for viral replication [36]. The genome of *Maculavirus* contains four ORFs, the longest (ORF1) encoding a replication polyprotein constituted by four domains: a methyltransferase (Mtr); a papain-like cysteine protease (P-Pro); an RNA helicase (Hel); and an RNA-dependent RNA polymerase (RdRp), which are conserved among all the ssRNA viruses [36, 37]. In the leaf sample evaluated in this study, a high number of contigs with similarity to the RdRp region was amplified by ddPCR, which may indicate that the putative new virus is active and replicating.

Although hundreds of plant viruses have been described over the past 120 years, only one species (*Carlavirus,* order Tymovirales) was found to infect native *H. brasiliensis* trees in Amazonia [11]. The virus occurrence was reported in leaves of stunted seedlings possessing inter-veinal chlorosis [11]. The genus *Maculavirus*, together with *Marafivirus* and *Tymovirus,* compose the *Tymoviridae* family (order Tymovirales). Members of the *Tymoviridae* family are usually associated with eudicotyledonous plants, which include *H. brasiliensis,* except for some *Marafivirus* that infect monocots (Poaceae). Infections by maculaviruses are known to be phloem-limited [36, 38, 39]. Our findings corroborate these data, since we only detected viral sequences in the leaves and not in the xylem tissue (sapwood).

## Conclusions

Small RNA deep sequencing combined with bioinformatic tools and in vitro amplifications proved to be a robust strategy for the accurate identification of viral sequences in *H. brasiliensis* leaf samples. Using this approach, we detected viral sequences associated to the rubber tree that could be ascribed to a putative novel virus, which was named *Hevea brasiliensis* virus (HBrV). As far as we know, this is the second time that viral sequences were identified in this plant; the first, *Carlavirus*, was described more than 30 years ago. Our results highlight the necessity of new studies to identify viruses associated with this economically important, but poorly studied plant.

## Additional files

Additional file 1: **Figure S1.** Overview of the strategy used for the in silico contig ordering. Contigs were anchored in the genome of related viruses based on sequence similarity searches. The position of contigs and the score associated to each position was stored for each reference genome assessed (partial score). After the evaluation of all references, the final contig position was defined by the highest cumulative score obtained through sum of partial scores. (PDF 422 kb)

Additional file 2: **Table S1.** Oligonucleotides designed for the amplification of viral contigs. (DOCX 59 kb)

Additional file 3: **Table S2.** Summary metrics of the sequencing and from the contigs assembled in sample C2. (DOCX 49 kb)

Additional file 4: **Figure S3.** Schematic representation of the Grapevine fleck virus (GFkV) organization. ORF1 (upper box) codes for the replication associated polyprotein (RP) containing the domains of methyltransferase (MTR); papain-like protease (PRO); helicase (HEL); RNA-dependent RNA polymerase (POL); and the coat protein (CP), ORF2 (lower box) encodes the putative movement protein (MP). Bars represent the contigs assembled according to the sequence similarity searches. Red arrows represent the oligonucleotides designed to amplify four fragments covering contigs regions across the genome. (PDF 399 kb)

Additional file 5: **Table S2.** DNA sequences corresponding to the viral contigs identified in the *Hevea brasiliensis* sample. (DOCX 119 kb)

Additional file 6: **Table S4.** Identification and characterization of the assembled contigs based on NCBI database searches. Similarity and coverage percentages, accession numbers, contig position and sizes are listed. (DOCX 92 kb)

## Abbreviations

AGO: Argonaute
AIC: Akaike Information Criterion
CNF: Caxiuanã National Forest
cPCR: Conventional PCR
ddPCR: Droplet Digital PCR
dsRNA: Double-stranded RNA
GFkV: Grapevine fleck virus
GOLD: Genome Online Database
HBrV: *Hevea brasiliensis* vírus
Hel: Helicase
Mtr: Methyltransferase
NCBI: National Center for Biotechnology Information
P-Pro: Papain-like cystein protease
PTGS: Post-Transcriptional Gene Silencing
qPCR: Quantitative PCR
RdRp: RNA dependente RNA polymerase
RISC: RNA-induced silencing complex
SALB: South American Leaf Blight
siRNA: Small interfering RNA
sRNA: Small RNA
TNF: Tapajós National Forest
vsiRNA: Virus small interfering RNA
