# Supplementary figures and images for: Virome analyses of Hevea brasiliensis using small RNA deep sequencing and PCR techniques reveal the presence of a potential new virus
Source: Virol J. 2018 Nov 26;15:184. doi: 10.1186/s12985-018-1095-3 (PMC6258436; doi:10.1186/s12985-018-1095-3)

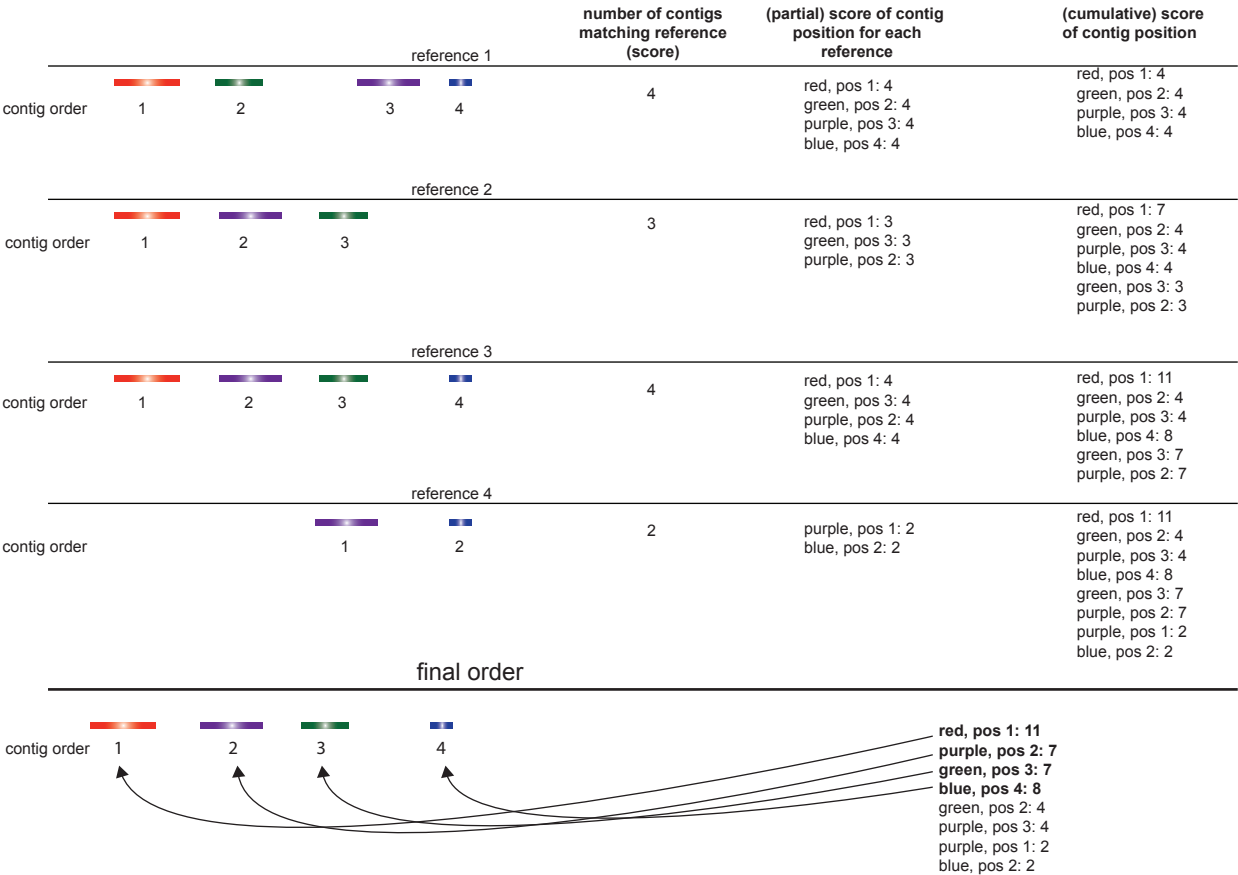

Supplement: Supplementary file 1 — Figure S1. Overview of the strategy used for the in silico contig ordering. Contigs were anchored in the genome of related viruses based on sequence similarity searches. The position of contigs and the score associated to each position was stored for each reference genome assessed (partial score). After the evaluation of all references, the final contig position was defined by the highest cumulative score obtained through sum of partial scores. (PDF 422 kb) [file 12985_2018_1095_MOESM1_ESM.pdf]

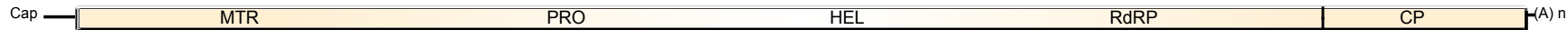

E-value:

■  $1e-2 < x < 1e-5$ 
■  $1e-5 < x < 1e-10$ 
■  $x < 1e-10$

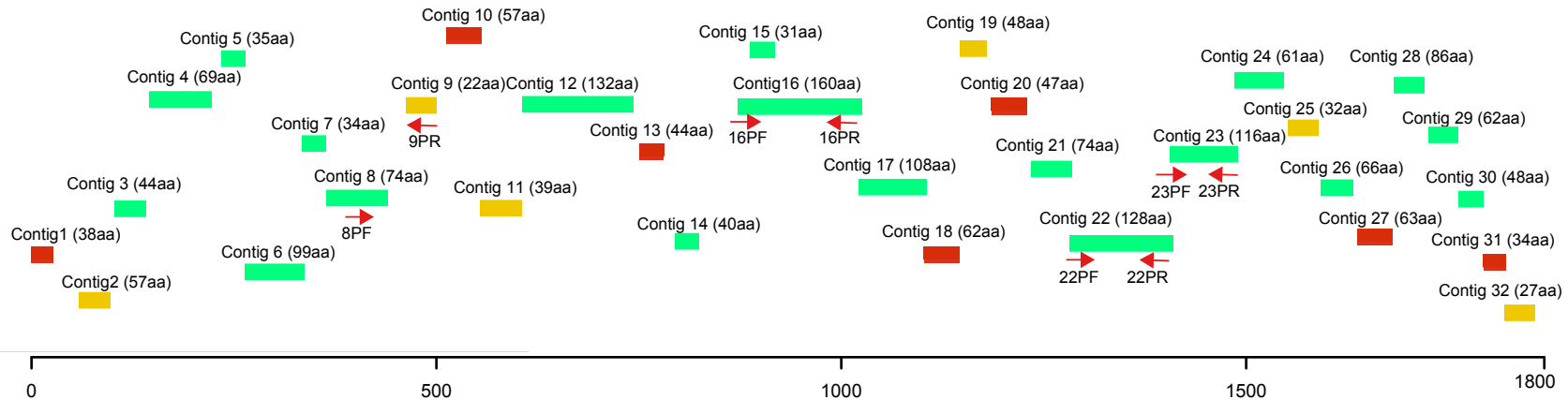

Grapevine fleck virus (GFkV) (aa)

Supplement: Supplementary file 4 — Figure S3. Schematic representation of the Grapevine fleck virus (GFkV) organization. ORF1 (upper box) codes for the replication associated polyprotein (RP) containing the domains of methyltransferase (MTR); papain-like protease (PRO); helicase (HEL); RNA-dependent RNA polymerase (POL); and the coat protein (CP), ORF2 (lower box) encodes the putative movement protein (MP). Bars represent the contigs assembled according to the sequence similarity searches. Red arrows represent the oligonucleotides designed to amplify four fragments covering contigs regions across the genome. (PDF 399 kb) [file 12985_2018_1095_MOESM4_ESM.pdf]
