# Supplementary material for: Virome analyses of Hevea brasiliensis using small RNA deep sequencing and PCR techniques reveal the presence of a potential new virus
Source: Virol J. 2018 Nov 26;15:184. doi: 10.1186/s12985-018-1095-3 (PMC6258436; doi:10.1186/s12985-018-1095-3)
Supplement: Supplementary file 2 — Table S1. Oligonucleotides designed for the amplification of viral contigs. (DOCX 59 kb) [file 12985_2018_1095_MOESM2_ESM.docx]

Additional file 2: Table S1. Oligonucleotides designed for the amplification of viral contigs.

| Contigs | Oligonucleotides sequences | | | PCR product (bp) |
| --- | --- | --- | --- | --- |
|  | **Foward (5`-3`)** | **Reverse (5`-3`)** | **Probe (5`-3`)** |  |
| Contig 23 | GGCTAGGTGAAGGGAATCGG | TGAAAAAGTATCAACGTGTGCTC | TGGCAGAATTCAGAGAGCTCAAATGGGG | 128 |
| Contig 8 and 9 | AGGGATCTGTGACACGAAGC | CTCCCCAACCCATCCACT | TTCGCAGACAGCCTTTGGGACTAA | 118 |
| Contig 16 | GGATGTGGCAAGTCCTACCC | GGACAGTTTCCACTCGGTTC | AGCAGCTTCTCCGTACCAAGCATT | 108 |
| Contig 22 | AGGCAGTCTGGAGTGAGGAG | TATCCAAAAGGCTCCGCTTC | AAAGACTGGTTCGTGGGCCAACTC | 102 |
