# Supplementary material for: Virome analyses of Hevea brasiliensis using small RNA deep sequencing and PCR techniques reveal the presence of a potential new virus
Source: Virol J. 2018 Nov 26;15:184. doi: 10.1186/s12985-018-1095-3 (PMC6258436; doi:10.1186/s12985-018-1095-3)
Supplement: Supplementary file 3 — Table S2. Summary metrics of the sequencing and from the contigs assembled in sample C2. (DOCX 49 kb) [file 12985_2018_1095_MOESM3_ESM.docx]

**Additional file 3: Table S2.** Summary metrics of the sequencing and from the contigs assembled in sample C2.

| Library | Total number of raw reads | Reads after quality and adaptor filtering | Number of contigs | N50 | Average | Median | STDEV | Largest contig | Number of viral contigs | Largest contig viral |
| --- | --- | --- | --- | --- | --- | --- | --- | --- | --- | --- |
| Lib01  (SRR6326570) | 10,755,497 | 9,324,929 | 303 | 67 | 69.755 | 63 | 24.072 | 260 | 15 | 260 |
| Lib02  (SRR6133884) | 10,690,564 | 9,266,320 | 282 | 66 | 70.808 | 61 | 30.042 | 328 | 16 | 328 |
| Merge | 21,446,061 | 18,591,249 | 110 | 136 | 128.442 | 106 | 61.696 | 483 | 32 | 483 |
