# Supplementary material for: Virome analyses of Hevea brasiliensis using small RNA deep sequencing and PCR techniques reveal the presence of a potential new virus
Source: Virol J. 2018 Nov 26;15:184. doi: 10.1186/s12985-018-1095-3 (PMC6258436; doi:10.1186/s12985-018-1095-3)
Supplement: Supplementary file 5 — Table S2. DNA sequences corresponding to the viral contigs identified in the Hevea brasiliensis sample. (DOCX 119 kb) [file 12985_2018_1095_MOESM5_ESM.docx]

**Additional file 5: Table S3.** DNA sequences corresponding to the viral contigs identified in the *Hevea brasiliensis* sample.

| >Contig1 GGTGGAACTGGGAGAAGGGGTGGATTCAGTGGATGAGGAGGATGGTTCAGTTGGGGGGGATTGGGAGGGACCAGAAGAGATGGAGGTGTGGATTGGGGAGACAGTGGGGGAGGGGG |
| --- |
| >Contig2  TCCTCTCCCACCTGTCTCCACTCACAACAACCTCCCACTTTCTCACATAATTCTCTTTCAGCTCTCACAATGGCCCTTCGCGCTGCTTTTGATTTCCTCCAGAACACCATCCACCGCGATACCATTGCCGCCACCATGGCTGCCAGCACCAACCCTGACTTCTCCCGCTCCCT |
| >Contig3  CCAGCTCGCTGGGTTCCCCTCCTCAATGACGCCGGTATCCCCGTCTCATCTCTTGGATTCCGCCCTCATCCACATCCCATCCACAAATCCATAGAGAACTTCCTCCTTTTCTCCCACTGGGCCGCTCGCGCT |
| >Contig4  CACTGAGAGACGAAGGGAGGATGGTGGAAGGGTCAGAGTCGGGGAAGCGATCATGATCGCGGGCGGTAATCCGAGGGTTGAGGAGGCGGGAGAAGAGAGGTGAATCAGAAGGATGGAGGGATTGCTGGGCGTCTTGAAGTTTGGCGAACTTTTCGGATTTCATCCAAAAGACGCAAGATGGTTCGGAAGCGCGAGCGGCCCAGTGGGA |
| >Contig5  CAAGGTGGGGGGCAGCGAGGAACAGACCGGCTATTTGGGCGGGGGAAATGTACATGAGCGCATCGTGGATGAAGTAGACACGCTCGGGGGGGGGAGACGAAGGGAGG |
| >Contig6 TGCCCCCTGAGGTCGCCATCGGGGAGCATTCCTTCTACCCCTCCATGTACCAATACCACTCCTCCGGGCTCAGACTTAACTACACTTTGGAGTCCAACCCCTCTTCCCACTATTCACAACCTCTCTCCGCTCAACAGTGGCTCTCCATAACCTCCATTCGAACCGAGAATCTCCACCTCACTGTCACAATCCTGGAGTCTTGGTTCAGTGTCCACTCCATCCTCATCACCCGCACACACCTTGAAGCTGACCTTCTCTTTGACACCATCGACTTCAAGGTGCCCTCCGCCATCCTCCTC |
| >Contig7  AGGTTTTTGGGCCTGAGTGCGAACGAAGCCGGAGGGATCTGTGACACGAAGCGTCCGAACGGCTCGGA |
| >Contig8 GGCCCAAAAACCTGAGTATGAATGGGTCACTTCGGAGGCCTGGGACAACCTAGCTCAGTTCGCGCTCCTCACTGCCCCTGTCCGCCCTAACACTTACTGGCTCTCCCTCACTTCCCCCTGGGCTGTCATCAGACACTGGCTCTTCCTCAACCAACGCCCAGTTCTTTCCTCCATCGCCGCCCTCACTTCACTCACTTTCTCAGCCATTTCCTACATTGTCACC |
| >Contig9  GCCGAACGGCTCGGACATAGATGAAAAGAGCTTCGCAGACAGCCTTTGGGACTAATCTGTGTCGCTCTGGGATGGTGACAGTGGATGGGTTGGGGAGGAGGATG |
| >Contig10  GAGAAGCGGCCACGGGGTCTGAGATCTTTGCGGAGAAATTGGTGGAAGAACTTGGGAAGGATGCGGGATGGTAAAGCCCGGAGGCGTTCAGTGAGGGTGGGAGTGTAGAATTTGTGGCCAAACAATGTGAAGTAGCAGGAGTGGGTTGAGCAAGAGGTGACAATGTAAGAAATGG |
| >Contig11  GGGGGAGAAGCGGGCGAAGAACAAATACCGCAGCCCGAGCAGGACAGGGAACACTGAAAAAGCGACCCCGGCATACAGCAGGACACGGTTGGGGCGGAGCGGTTTAAGGAGCCAGGTGGG |
| >Contig12  TCCCCCGTCTCCGCCTCCATCGACCCCCCTCCATCTGCTCCCGCCCGGCTCTGGTGTGAAGTCTTCCCAGGCTCATACCTCGACCGAAGCGGCGAGTTCATCTTCCATCAAAGGATCACCCCCCCCTCCAATCTCCCCATCCCCACCAATGACTGTCTTCTCCGCGCCTGCTCCCATGCCACCAACATATCAGTCCTCACCCTATGGGAGGCTCTTGGTTCCTTCTTCACTGATAGGGACCTCGATGCCTCCTGTGGGCTCAACACCGACCACCTCACCGCTCTCTCCTTCATCTTTCGCTGGGTCGTCACCGTCTATCATGATGGTGGCTCCCAGCGCCATGGTCTCGACCTCCCTGATGCCCTCCACTTCTCCATCCAGCACTCCCCTCCCATTCC |
| >Contig13 CTTCTTCTCGTCCCTCCCGGCCTTCCAGGCCTTCCAGGGATCTAACTGACTCTCCAACTCCCTCCCCCTCCCACTCCCAATTAGCCCGCCGCCTCCTTTCCTTCCGCATCAACCGTGAACCACTCCCTGTCACC |
| >Contig14 CCAAAAACCTGGCTTCCAACATGAAGAACGGACATGATGGTGTCCTCTCCTCCCTTTCCTCCGCTGACCGCATTCAAGCCGCTAACTACATCAAATCCCTTGACAACTCCGCTGACTTCCCC |
| >Contig15  AGTCCTTCTGTCGGGCTTGTCCACCTTGCCGGTTTCCCCGGATGTGGCAAGTCCTACCCTGTGCAGCAGCTTCTCCGTACCAAGCATTTCTCTCA |
| >Contig16  AGTCCTTCTGTCGGGCTTGTCCACCTTGCCGGTTTCCCCGGATGTGGCAAGTCCTACCCTGTGCAGCAGCTTCTCCGTACCAAGCATTTCTCTCATTTTAAGCTAGCTGTCCCCACTTCTGAGCTTCGAACCGAGTGGAAACTGTCCATGGCTCTGCCCAACACAGAGTCGTGGCGGTTTTCCACTTGGGAGGTCTCCCTACTGAAGCGAGCGAAGATTCTCGTCATTGACGAGGTCTACCGCTTGCCAAGGGGGTATCTTGACCTCTGCTTGCTCTCGGACGCCACAATCGAGCTCGTCATTCTGCTCGGCGATCCGTTACAGGGTTCCTACTTCCCCACCTCCCCTGACTCATCCAACCTCCGGCTCACTCCTGAAGAGGACCATCTCCGCCCTTACATCGACTTCTACTGCATGTGGTCCCGCCGGATTCCCCAAAACCTAGCCTCCTTTTTCCAAGTCCAATCCACCAACCCCGCCC |
| >Contig17  GCGGCGGCTTGGGAGGTGTAGTTGTGGAAGAGAGTGAAGAGGTGGTCTCGGGATTTTGGGAGGTGGTTGGTGTCGGGGATCCAGATGATGCCAGCCTTCGTGCGGGTGAGCGCGACGAGGGCGACATGGTTTGAGACAAGGGCGGTGTGGTTGGACAGGAGGATGGAAGCTGGACCCTGGAAGGTTGAGCCTTGGGAGGAGGCATATGTGATAGCGCGGTGACCAGTGGAGGAAGTGGTTTTGGCAGCGGAGTCAGAGGGGACAAGGAGAGGGGACCTGGAAGGGTTGAGGGAGCCTTTGGATACGAATCCTGGGGCGGGGTTGGTG |
| >Contig18  ACATCTCTCAAACCCTCATCATCCCAAACACACGCCCCCTTCCAAGCCATGCCCCGTTTCCCAAAACCTACCCCCCGTGTCTACACCACTTTCCCTGACTCTCTCCGCCACCAACTGCACCTGCCCTCTCACTCCCACAACCTAGCCAGAACCATTTCATCAGCCCACCCATCAGCCAACTCCCTTC |
| >Contig19  AGAGGACAGGGGGGAGGGGTGAAGGTCGGTGGGGGGGAACCTGACAAGGAAGAGCTGGGAGGGATCCCGCAGAATTCTCAGACCAGCAAGCTGGCGCTGGAAGACTACGGTGAGATTGCAGGGTTGGGGAGGGGGTTGGGTGGAG |
| >Contig20  GAGCGGGTGGGGCGAGAGATGGAAGTTGGAGGATGTGGGGTTGGAGCTTGGTGGCATTTAATGGATATGGGATGGATGGTCTGTGAGGGAATTTGACTGGACGGGCACCAGAGAGGCGGGAACAGGATTTGAACCAGCGTAGA |
| >Contig21  CTCCCTTCTCCCCACCCTACCCCTTGATCAAGCCACATCCTACCCGACCTCCGCCCTTCCCCATCGCGTTTTCGCCCCTGCCCTCACGGATTTTCCTGAGGAAGAAGGCGATCTTCGTGTCAACCCCACCTTTGTAGCCGAAACCCGTCTCCCCTTCCAAACTGACCTTGCCCCTTCTAACATATCATCTGCTGACCCTTCTGACACCCCCATCTCCACCACC |
| >Contig22  TGGTCCGAGAACCAGGATCAAGTAGTCATGCATGAGCGCGAGGGTTTGACAGGCCTTCCAACCGCTGAACAAAGAATTCTCATTGACCTTATGCTGGGTCTTTGAGAAAATCCGAACAGCGGAGTACCTCCAGTCTGGATCAGAACGATGGGAGTTGGCTTGGATGACGGCTTGAGATTTGGAAGAGAGCTGGGCCCAGTCATTGGCATTGATGCAGTTTGAGAACAGGACCGGGTCGAATGGAACAGGGTCTAGAGAACGGCGGTAGGCAGTCTGGAGTGAGGAGAAGAGGAGTTGGCCCACGAACCAGTCTTTTTCGGAGAGCTGGTAAGGGGCGTCAGACTCGCGGAAGCGGAGCCTTTTGGATATGGCGGATGGGAGCAGGG |
| >Contig23  CTGTAAAGAACAGCCAGGTTGTAATCGGTGTTATCGTCGTAAGTCCCGGGTTCACCCGTTAGACGCATGCAGGTGAGGGGGCCAAATTGGGTAGTGATGGAGGTTTTGATGTGTGAGTGGAGGGAAATGAGGGAGTCAGGAATTGAGCAACGGCGCATTTTCAGAATTTCAAGGACAAGCGCTTCGCCGTGCTGGCTTTGATCAAAGGAGGTGTAGTCGTTGGCTAGGTGAAGGGAATCGGTCATGAAAGATTGGCAGAATTCAGAGAGCTCAAATGGGGTGTGGGAGGCATGGACGTAGAGGTGTGGGGGCCTGTCACGACCATCGAGCACACGTTGATACTTTTTCACT |
| >Contig24  TACAGCCAGTTTGTACTCGGTGATATCGCCGTCCTTGTCTCTGGCGATGACTCCCTCGTAGACCGTGTCCCAGCCCCAAACCCCATCTGGTCTTTCCTCGGTCCACTCCTCTCCCTCAAGCCCAAACCGGAGATCTCAGCGTACGGCCTCTTCTGCGGCTACTACGTTGGGCCCGCCGGTGCCA |
| >Contig25  CTCTCTTCACCAAACTCATGATTGCTTACCAGACTAACAACCTCCCCAAAGTCCTAGCCTCCTACATAGCTGAGTTTTCTGTCGGCCATTCCCTCGGCC |
| >Contig26  GAAGGCCGCTCGGGCCCTGGAAGAAAGCATGGAAAACAGTGGGCGGGAGACAAAACGGGTTTCAATGGAGTTGGCGAGCTCGGCCCAGTCAGGGACTTCGCCGAGATGGAGGGCGACTTTGAGGTTCTTGGAGGCATGTTGACAGATGAAGGAATTGAAGAACAAATACCGCAGCCCGAGCAGGACAGGGAACACTGAA |
| >Contig27  TTTTCAATATCTACCAACCTGACTCCTGCTTTCGCCCTTCTGCTCCTCCTGTCCTCCTTCCTCACTGTTTGTCTTCTCCTAGCCATTTGTGCCCTTTATCGTGTCGCCCTCCATCGCCTGTCTTCCTCCACCGCTGTCCTAACCAGTCCGAATCCAGCCCAGGTCCTCCACTATCCTGCCCACCTCCCAC |
| >Contig28  TCCTCCCCTTCCAATTCAACTGGTTCGATATCACCGGTGTCGAAACCAAACACGCTTCCCTCGATGTTGCCTCCCGCCCTGAACTCCGAGCCCTCGCCAAACCCTACCGCCATGCCAAAATCCGAACTCTCGAAGCCACTGTCATGCCCACAGCCCCCTCTGCTACCTACCCCCAAACCGTCGAACTCTGCTGGACCATCTCCTCCATCACGCCCGCTGAAACTGAAATCATCACTGTCTACGGCGCTCAACGCATTGCGT |
| >Contig29  GTGGGACCGGCTGAGGGGGCGGAGAGTTCGAGCGTGCCGCGAACAATGATGGAGCCGCAGATGACGGCACTGGTTTTGAGAGCTTCGCAGGCAGCATTGGGGAAGAACTTGGCGGTGAGGCGGGGAGTGTCGGTGTAGGTGACTGAGTCTTTGACAACTGGGTTGAGGTGGGTGAGGTCGGCTGGGAG |
| >Contig30  TTGGCACCATCAGATTCACATTCGGGAACTGAGTTTCCTTGCTCGTTCCCAGTATAGCAATATTCCCTATCAGATAATCCAGGTAGTGATTCAGAATAGCCCTCTTGGTTGTCAATTGATTCTGACTCACCACTGGATTCCCCTC |
| >Contig31  TGAATATTCCTGTTGAATTGGTGGAGGAATATATTCAATTGAAAACGCATTTGGACAGTAAATTAGGGACATTGGCAATAATGAGATTTACTGGTGAATTTGCC |
| >Contig32  TCTTCAAACACAGCTTTCTGACCCTCAGTCCTAAAGAGAGAACTGTTTAGGGCTGAGACTACTTGAGCCCTAGCATCATTAAG |
