# Supplementary material for: Virome analyses of Hevea brasiliensis using small RNA deep sequencing and PCR techniques reveal the presence of a potential new virus
Source: Virol J. 2018 Nov 26;15:184. doi: 10.1186/s12985-018-1095-3 (PMC6258436; doi:10.1186/s12985-018-1095-3)
Supplement: Supplementary file 6 — Table S4. Identification and characterization of the assembled contigs based on NCBI database searches. Similarity and coverage percentages, accession numbers, contig position and sizes are listed. (DOCX 92 kb) [file 12985_2018_1095_MOESM6_ESM.docx]

**Additional file 6: Table S4.** Identification and characterization of the assembled contigs based on NCBI database searches. Similarity and coverage percentages, accession numbers, contig position and sizes are listed.

| Contig number | Identification | Coverage (%) | Identity (%) | Acession Number | Query Size (aa) | Frame | Contig Size (aa) | Region | Genus | ORFs acession number (aa) |
| --- | --- | --- | --- | --- | --- | --- | --- | --- | --- | --- |
|  |  |  |  |  |  |  |  |  |  |  |
|  |  |  |  |  |  |  |  |  |  |  |
| Contig 2 | Grapevine fleck virus | 57 | 48 | ADM53707 | (+1)162 | 1 | 57 | Replicase | Maculavirus | NP_542612 |
|  |  |  |  |  |  |  |  |  |  |  |
| Contig 3 | Grapevine fleck virus | 100 | 64 | ADM53704 | (+1)162 | 1 | 44 | Replicase | Maculavirus | NP_542612 |
|  |  |  |  |  |  |  |  |  |  |  |
|  |  |  |  |  |  |  |  |  |  |  |
| Contig 4 | Citrus sudden death-associated virus | 83 | 43 | AQM73488 | (-1)203 | -1 | 69 | Methyltransferase | Marafivirus | NC_002786 |
|  |  |  |  |  |  |  |  |  |  |  |
| Contig 5 | Grapevine fleck virus | 98 | 63 | NP_542612 | (-2)1949 | -2 | 35 | Replicase | Maculavirus | NP_542612 |
|  |  |  |  |  |  |  |  |  |  |  |
|  |  |  |  |  |  |  |  |  |  |  |
| Contig 6 | Grapevine fleck virus | 99 | 57 | CAC10496 | (+3)191 | 3 | 99 | Methyltransferase | Maculavirus | NP_542612 |
|  |  |  |  |  |  |  |  |  |  |  |
| Contig 7 | Bombyx mori Macula-like virus | 97 | 95 | BAD35017 | (-3)1747 | -3 | 22 | RNA-dependent RNA polymerase | Maculavirus | BAD35017 |
| Contig 8 | Grapevine fleck virus | 87 | 63 | NP_542612 | (+2)1949 | 2 | 74 | Replicase | Maculavirus | NP_542612 |
| Contig 9 | Grapevine fleck virus | 98 | 71 | NP_542612 | (-2)1949 | -2 | 34 | Replicase | Maculavirus | NP_542612 |
| Contig 12 | Grapevine fleck virus | 88 | 43 | NP_542612 | (+1)1949 | 1 | 132 | Replicase | Maculavirus | NP_542612 |
| Contig 14 | Grapevine fleck virus | 98 | 55 | NP_542612 | (+3)1949 | 3 | 40 | Replicase | Maculavirus | NP_542612 |
|  |  |  |  |  |  |  |  |  |  |  |
| Contig 15 | Grapevine fleck virus | 91 | 72 | NP_542612 | (+1)1949 | 1 | 31 | Replicase | Maculavirus | NP_542612 |
| Contig 16 | Grapevine fleck virus | 98 | 73 | NP_542612 | (-2)1949 | -2 | 100 | Replicase | Maculavirus | NP_542612 |
| Contig 17 | Grapevine fleck virus | 94 | 53 | NP_542612 | (-2)1949 | -2 | 108 | Replicase | Maculavirus | NP_542612 |
| Contig 19 | Grapevine fleck virus | 57 | 57 | NP_542612 | (-2)1949 | -2 | 48 | Replicase | Maculavirus | NP_542612 |
| Contig 21 | Grapevine fleck virus | 87 | 58 | NP_542612 | (2)1949 | 2 | 74 | Replicase | Maculavirus | NP_542612 |
| Contig 22 | Grapevine fleck virus | 99 | 71 | NP_542612 | (-3)1949 | -3 | 128 | Replicase | Maculavirus | NP_542612 |
| Contig 23 | Grapevine fleck virus | 96 | 76 | AEK10706 | (-2)118 | -2 | 116 | Replicase | Maculavirus | NP_542612 |
| Contig 24 | Fig fleck-associated virus | 99 | 69 | YP_004300278 | (+1)2161 | 1 | 61 | Polyprotein | Maculavirus | NP_542612 |
| Contig 25 | Culex originated Tymoviridae-like virus | 96 | 69 | AGE84283 | (+3)1761 | 3 | 32 | Replicase | Maculavirus | NP_542612 |
| Contig 26 | Grapevine fleck virus | 72 | 52 | NP_542612 | (-2)1949 | -2 | 66 | Replicase | Maculavirus | NP_542612 |
| Contig 28 | Grapevine fleck virus | 98 | 67 | AEP27093 | (+3)160 | 3 | 86 | Coat Protein | Maculavirus | NP_542612 |
| Contig 29 | Grapevine fleck virus | 98 | 63 | ADM53708 | (-1)224 | -1 | 62 | Coat Protein | Maculavirus | NP_542612 |
| Contig 30 | Cherry virus A | 91 | 43 | ARQ83948 | (-1) 2342 | -1 | 48 | Polyprotein | Capillovirus/Betaflexviridae | AOG18264 |
| Contig 31 | Cherry virus A | 95 | 61 | AOG18261 | (+3)78 | 3 | 34 | RNA-dependent RNA polymerase | Capillovirus/Betaflexviridae | AOG18264 |
| Contig 32 | Mint virus 2 | 93 | 58 | AAX07262 | (-1)209 | -1 | 27 | Coat protein | Vitivirus/Betaflexviridae | AY913795 |
